# Primary gastric tuberculosis presenting as gastric outlet obstruction: a case report and review of the literature

**DOI:** 10.1186/s13256-015-0748-8

**Published:** 2015-11-18

**Authors:** Nassir Alhaboob Arabi, Abdulmagid M. Musaad, Elsaggad Eltayeb Ahmed, Mohammed MAM Ibnouf, Muataz Salah Eldin Abdelaziz

**Affiliations:** 1 Department of GI Surgery, Ibn Sina hospital, Khartoum, Sudan; 2 Department of GI surgery, Ibn sina Specialized hospital, Khartoum, Sudan

**Keywords:** Abdominal tuberculosis, Gastric outlet obstruction, Gastric tuberculosis

## Abstract

**Introduction:**

Tuberculosis is a major health problem worldwide. Sudan has high burden of tuberculosis (TB) with a prevalence of 209 cases per 100,000 of the population and it is commonly presented with pulmonary disease but involvement of the gastrointestinal tract is not uncommon. Abdominal tuberculosis comprises about 1–3 % of all cases of tuberculosis and about 12% of extrapulmonary tuberculosis. It involves the ileocecal region, but involvement of stomach and duodenum are rare sites. Here we present an unusual case of gastric outlet obstruction due to gastric tuberculosis.

**Case presentation:**

A 54-year-old Sudanese man presented with a non-bile stain persistent projectile vomiting, and epigastric pain for two years associated with marked loss of weight. There is no fever or cough. He was on antacid, physical examination showed BMI 18 and stable vital signs. He was not pale or jaundiced, there was no cervical lymphadenopathy and chest was clear. Abdominal examination was normal apart of positive succussion splash. The results of haematological tests were normal, ESR was 30 mm/hr, hepatitis B, C and HIV were negative. Upper gastrointestinal endoscopy showed that the stomach was full of fluid and food particles and ulcerated mass in the pylorus extended to the proximal part of the duodenum with severe narrowing of the pylorus. The lesion biopsied and the result revealed active inflammatory cells, cryptitis and multiple lymphoid follicles, no malignancy seen. Sonographic test showed hypodense pyloric mass, enlarged para-aortic and mesenteric lymph nodes and mild pelvic ascites. A computed tomography scan of the abdomen and pelvis showed antral hypodense lesions multiple mesenteric lymphadenopathies peritoneal thickening and ascites. Chest X-ray was normal. Intra-operative findings were dilated stomach and pylorus mass with multiple mesenteric lymph nodes, peritoneal and omental seedlings all over with small nodules on the surface of the liver, gastro-jejunostomy was done. Histopathology confirmed the diagnosis of abdominal tuberculosis. Postoperative event was uneventful. Patient received anti-tuberculous.

**Conclusions:**

Here we presented an unusual case of gastric outlet obstruction due to primary gastric tuberculosis, patient underwent surgery to relief his symptoms and received anti-tuberculous.

## Introduction

Tuberculosis (TB) is a major health problem worldwide and during 2008 there were 8.9 to 9.9 million reported cases of TB all over the world, most of them in Africa and Asia [1]. Sudan has a high burden of TB with a prevalence of 209 cases per 100,000 of the population and 50,000 incident cases during 2009 [2]. Pulmonary TB accounted for 73.4 % of all patients with TB in Sudan while extrapulmonary TB accounted for 26.6 %. In Sudan, patients with TB usually had less education than average, and more male patients than female patients were infected [3]. Involvement of the gastrointestinal (GI) tract is not uncommon and it often involves the ileocecal region [4]. Abdominal TB comprises approximately 1 to 3 % of all cases of TB and approximately 12 % of extrapulmonary TB [5]. The stomach and duodenum are rare sites for TB and are usually a result of secondary spread from a primary pulmonary disease. A series by Rao *et al.* reported an incidence of gastroduodenal TB of only 0.5 % [4] and isolated gastric TB without evidence of a lesion elsewhere is even rarer [6]. Duodenal and gastric TB were found in only 1 % of patients with pulmonary TB with associated human immunodeficiency virus (HIV) infection in non-endemic areas; duodenal obstruction due to TB is very rare and needs a high index of suspicions for diagnosis [7]. The pyloric stenosis resulting from TB is even rarer than gastroduodenal TB. This, however, should be considered in patients who come from areas where the disease is endemic [8]. On clinical examination gastric TB resembles peptic ulcer disease or malignancy and it may be difficult to distinguish; the possible routes of infection include direct infection of the mucosa, hematogenous spread, extension from neighboring tuberculous lesion [9] or secondary to pulmonary TB [10]. A few cases of primary gastroduodenal TB were reported in the literature [11]. Primary gastric TB is usually a diagnostic challenge and may mimic peptic ulcer disease and even a neoplasia [12] or other conditions, including inflammatory bowel disease, malignancy and other infectious diseases [13]. The reason for this rarity is attributed to the bactericidal property of gastric acid, scarcity of lymphoid tissue in the gastric wall and intact gastric mucosa of the stomach [14, 15]. Gastroduodenal TB has three forms of presentation: obstruction, upper GI bleeding, and gastric or periampullary mass suggestive of malignancy [4].

## Case presentation

A 54-year-old Sudanese man presented with persistent non-bile-stained projectile vomiting and epigastric pain for 2 years associated with marked loss of weight. He had no jaundice, fever or change in bowel habits. He did not have a cough or hemoptysis and his other systems were unremarkable. He had no significant past medical history, no history of TB, HIV infection or diabetes, he was not hypertensive, and there was no family history of a similar condition or TB. He was on antacid medicine; he was not a tobacco smoker and neither was he an alcoholic. A physical examination showed a body mass index (BMI) of 18, normal vital signs, he was not pale or jaundiced, there was no cervical lymphadenopathy and his chest examination was clear. His abdomen was flat, moved with respiration, with no dilated veins, surgical scars or cautery marks and hernia orifices were intact. There was no tenderness, masses, organomegaly or ascites; his succussion splash was positive. The results of hematological tests were normal, his erythrocyte sedimentation rate (ESR) was 30 mm/hour, and hepatitis B, C and HIV were negative.

**Fig. 1:**
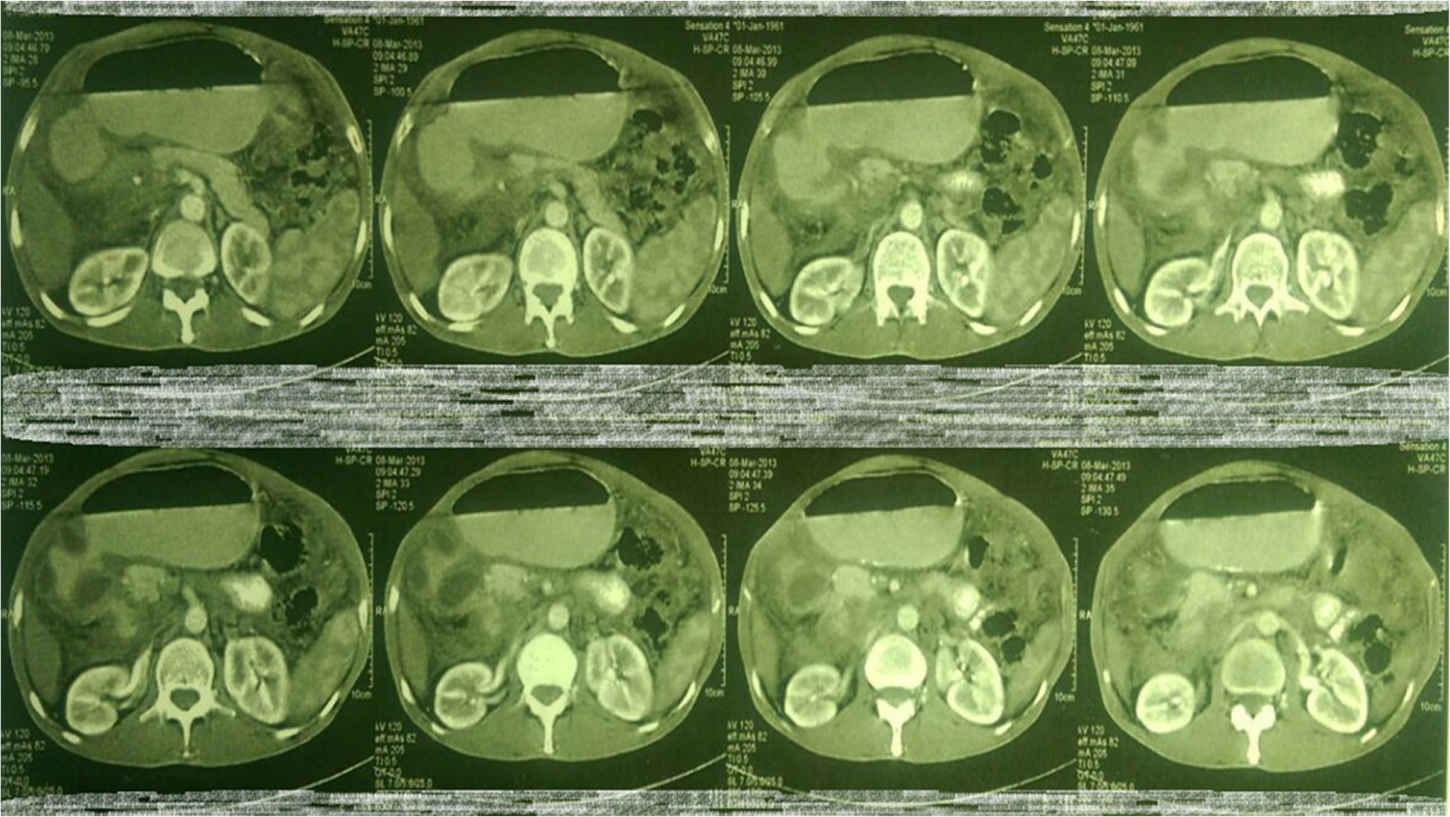
A computed tomography scan of the abdomen showed nodular hypodense lesions surrounding the antrum of the stomach with gastric dilatation, multiple mesenteric lymphadenopathies and peritoneal thickening and ascites

**Fig. 2:**
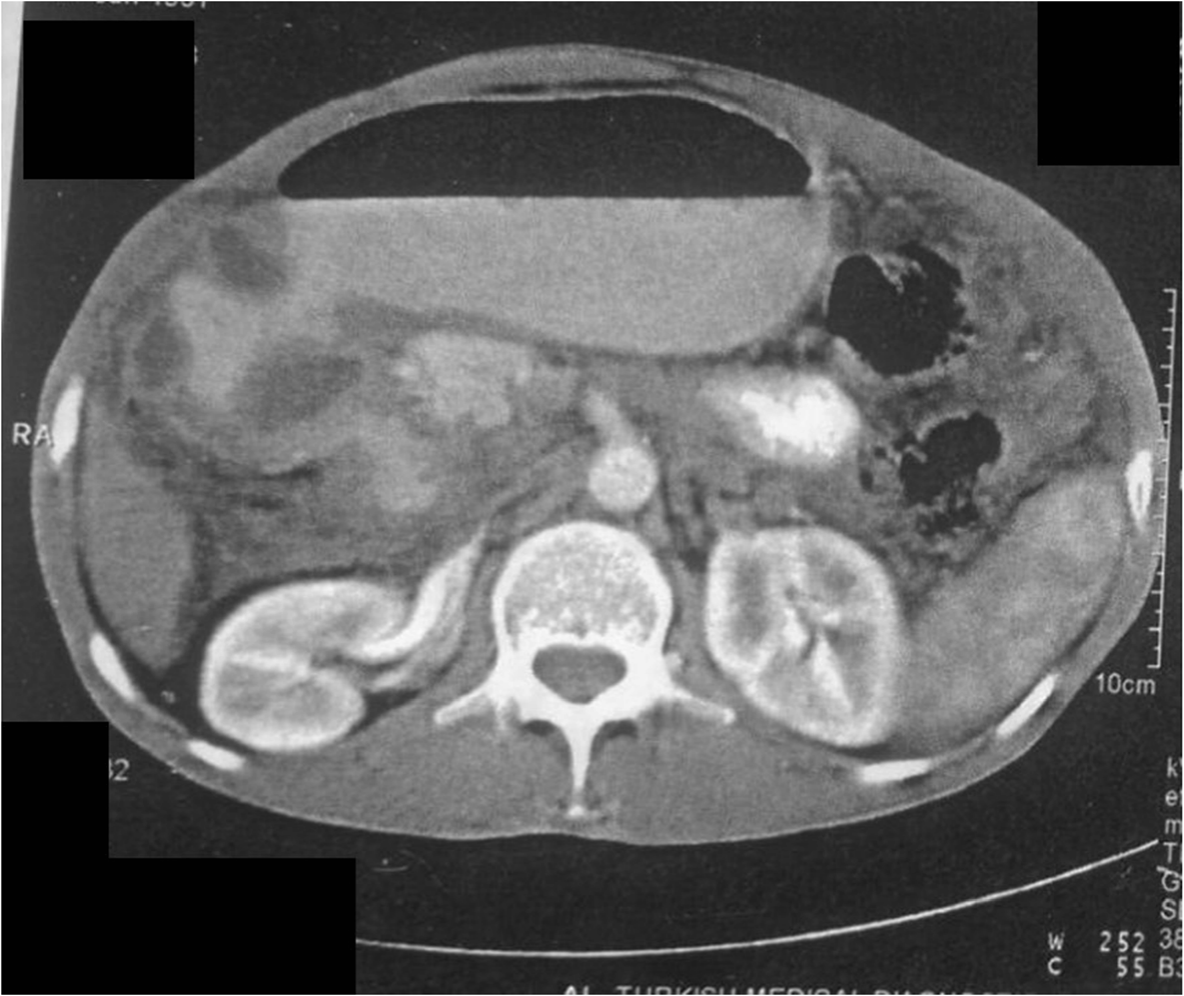
A computed tomography scan of the abdomen showed nodular hypodense lesions measuring 30 mm surrounding the antrum of the stomach with gastric dilatation, and multiple mesenteric lymphadenopathies measuring 40 mm

**Fig. 3:**
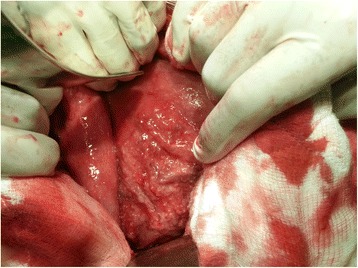
Intraoperative finding: omental and mesenteric seeding

**Fig. 4:**
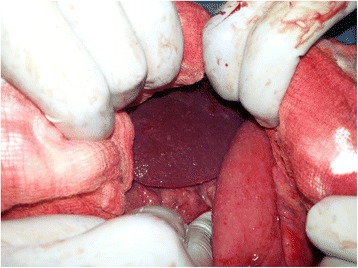
Intraoperative finding: liver seeding

**Fig. 5:**
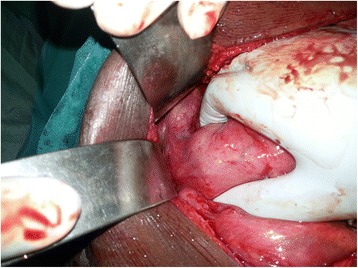
Intraoperative finding: the pyloric mass

**Fig. 6:**
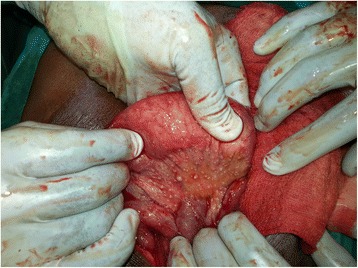
Intraoperative finding: small bowel and mesenteric seeding

**Fig. 7:**
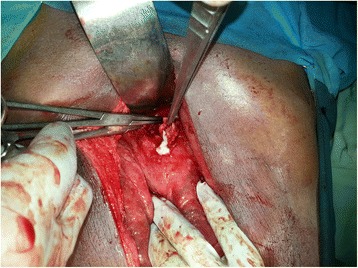
Intraoperative finding: caseating material after dissecting one of the lymph nodes

**Fig. 8:**
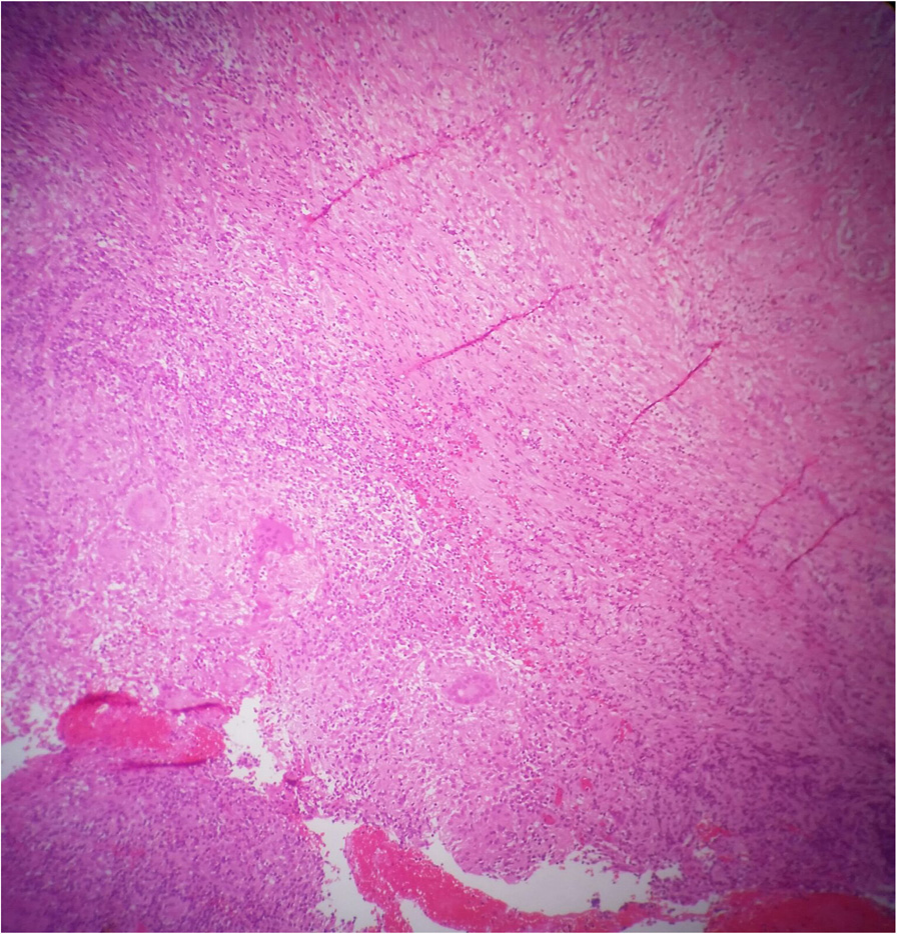
Histopathology: caseating granulomas with lymphocytes

An upper GI endoscopy showed that his stomach was full of fluid and food particles and an ulcerated pyloric mass extended to the proximal part of his duodenum with severe narrowing. Multiple biopsies were taken and histopathology revealed gastric mucosa heavily infiltrated by florid active inflammatory cells disrupting the glands, which consisted of neutrophils, lymphocytes and plasma cells. The glands exhibited cryptitis and regenerative changes with the presence of multiple lymphoid follicles. No *Helicobacter* *pylori*, dysplasia or evidence of malignancy was seen. A sonographic test showed a 4^.^4×2^.^5 cm hypodense focal soft tissue mass in his pyloric region with enlarged para-aortic and mesenteric lymph nodes, there was minimal pelvic ascites, normal liver and other organs. A computed tomography scan of his abdomen and pelvis showed nodular hypodense lesions measuring 30 mm surrounding the antrum of his stomach with gastric dilatation and multiple mesenteric lymphadenopathies measuring 40 mm. Peritoneal thickening and ascites were also noted, otherwise, he had a normal liver, spleen, pancreas, kidneys, pelvic organs as well as aorta and inferior vena cava (IVC; Figs. 1 and 2), and a normal chest X-ray. A decision was made to relieve the obstruction. Intraoperative findings were: dilated stomach and 8×7 cm mass at the gastric pylorus with multiple mesenteric lymph nodes, and peritoneal and omental seedlings all over with small nodules on the surface of the liver; a gastrojejunostomy was done with multiple biopsies from the mass and the lymph nodes which showed caseating material during dissection (Figs. 3, 4, 5, 6 and 7). The result of histopathology confirmed the diagnosis of abdominal TB (Fig. 8). The patient’s postoperative course was uneventful and he started feeding on day four; he was discharged in good condition. Moreover, he was on serial follow up which showed that he gained weight of more than 1 kg over 20 days. He was referred to the TB eradication program for antituberculous therapy and screening for pulmonary TB which was negative (acid-fast bacilli, AAFB).

## Discussion

Although abdominal TB can develop at any age, it is most common in patients between 25 and 45 years of age and females slightly predominate [16]. Patients with gastroduodenal TB can present with obstruction or mass and an endoscopic biopsy has a poor yield [4]. Gastric lesions typically cause dyspeptic complaints, and generally, peptic ulcer is suspected. If the patient has lost weight, in addition to these complaints, gastric cancer should be considered first [17]. Gleason *et al.* reviewed 49 patients with duodenal TB; they found that the most common presenting symptoms were pain (73 %) and vomiting (55 %), whereas GI bleeding was rare (16 %) [18]. A report by Chetri *et al.* described a case of gastric TB presenting as non-healing gastric ulcer and out of five cases, three presented with gastric outlet obstruction, which is the most common presentation of gastric TB [19]. It may present as multiple shallow ulcers, especially on the lesser curvature of the stomach [20], or as a nondescript hypertrophic submucosal mass [21]. Another study showed that long-term therapy with H2 blockers increases the incidence of gastroduodenal TB [22]. In investigations of patients, a chest X-ray may show evidence of pulmonary TB in up to 20 % of cases [23] and upper GI endoscopy may reveal duodenal bulb deformity [24]. Endoscopic biopsy has a poor yield even in ulcerated lesions and endoscopic biopsy rarely reveals granulomas because of the predominantly submucosal location of these lesions and the failure of routine endoscopic biopsies to include the submucosa [17]. The diagnosis of duodenal TB is usually made after surgical intervention (exploratory laparotomy) and it is very rarely made preoperatively [25]; however, Sharma *et al.* reported that endoscopic ultrasonography (EUS) is an excellent modality for characterizing the lesion, as well as obtaining a sample for cytological confirmation of the diagnosis [26]. Multiple intraoperative fine-needle aspiration cytology (FNAC) may be taken from the diseased portion of the duodenum to establish the histopathological diagnosis if not established by any other means [7]. When the diagnoses of TB are established before surgery, most lesions regress with appropriate antitubercular treatment and do not require excision [27, 28]. Minimally invasive procedures such as laparoscopic, endoscopic and percutaneous biopsy should be used for diagnosis of intraperitoneal TB as a first step in diagnosis, and laparotomy should be performed only when complications develop or diagnosis remains unclear in spite of these diagnostic modalities [16]. Surgery is usually required for diagnosis or therapy, after which patients respond well to antituberculous treatment. In areas endemic for TB, a good biopsy from the site of gastroduodenal bleeding or mass lesion and the surrounding lymph nodes should always be obtained [4]. In patients with gastric outlet obstruction, gastrojejunostomy is preferred over pyloroplasty, as intense fibrosis around the pyloroduodenal junction precludes safe pyloroplasty [29]. Puri *et al.* showed that endoscopic therapy in combination with antituberculous therapy is recommended as the first-line therapy for gastroduodenal TB and surgical intervention is reserved for the minority in whom endoscopic therapy fails [30].

## Conclusions

Primary gastric TB is rare, it is usually a diagnostic challenge and may present with gastric outlet obstruction. It should be suspected in TB-endemic areas. Surgery is often required for diagnosis and therapy.

## Consent

Written informed consent was obtained from the patient for publication of this case report and accompanying images. A copy of the written consent is available for review by the Editor-in-Chief of this journal.

## Abbreviations

GI: Gastrointestinal
HIV: Human immunodeficiency virus
TB: Tuberculosis
